# Beyond Enteric Infections: A Case of *Salmonella* Spinal Epidural Abscess—Case Report

**DOI:** 10.1155/crdi/6378392

**Published:** 2025-11-20

**Authors:** Ijeoma Ikedum, Mexan Mapouka, Folasade Arinze

**Affiliations:** ^1^Internal Medicine Department, Graduate Medical Education, WellStar Kennestone Regional Medical Center, Marietta, Georgia, USA; ^2^Internal Medicine Department, Graduate Medical Education, University of Maryland Capital Region Health, Largo, Maryland, USA

**Keywords:** discitis, epidural abscess, *Salmonella*, vertebral abscess, vertebral osteomyelitis

## Abstract

Spinal epidural abscess (SEA) is a serious but rare condition that is often challenging to diagnose early due to atypical presentations, especially in patients without common risk factors such as immunosuppression or sickle cell disease. This case report describes a 49-year-old woman with a history of alcohol use disorder who presented with flank pain and other nonspecific symptoms, initially misdiagnosed as musculoskeletal pain. MRI eventually revealed a SEA caused by nontyphoidal *Salmonella* (NTS), requiring surgical intervention and antibiotics. The discussion highlights the difficulty of diagnosing SEA, especially in patients without classic risk factors, emphasizing the need for high clinical suspicion, thorough history-taking, and timely use of MRI. Prompt imaging, neurosurgical consultation, and antibiotic administration are critical for effective management and favorable outcomes. Strategies for improving early diagnosis include using decision guides for emergent MRI and repeat imaging if initial results are nondiagnostic. This article aims to provide clinicians with an overview of SEA caused by NTS, emphasizing the importance of maintaining a high index of suspicion, critically examining reported risk factors, and exploring potential approaches for earlier diagnosis.

## 1. Introduction

Spinal epidural abscess (SEA) is an uncommon but serious condition associated with significant morbidity that often eludes early diagnosis. According to Strohecker and Grobovschek, “the problem with SEAs is not treatment, but early diagnosis, before massive neurological symptoms occur” [1]. It traditionally presents as a triad of back pain, fever, and neurological deficits [2–4]. However, early diagnosis is often challenging due to atypical presentations in most cases. *Staphylococcus aureus* is the most common cause of SEA and accounts for about two-thirds of cases caused by pyogenic bacteria [2]. Gram-negative bacilli are less commonly isolated. *Salmonella*, a gram-negative bacillus, causes infections that can be categorized into two groups: those leading to enteric fever (caused by *S. Typhi* and *S. Paratyphi*) and nontyphoidal *Salmonella* (NTS) infections, which primarily result in gastrointestinal illness. Enteric fever, caused by typhoidal strains, typically presents with sustained fever and systemic symptoms over a longer duration, unlike acute gastroenteritis and watery diarrhea commonly seen in NTS infections. Enteric fever has an average incubation period of 14 days, with symptoms persisting for up to 3 weeks. In contrast, NTS infections usually manifest within 6–12 h and resolve within 10 days, unless there is an underlying immunodeficiency, which can lead to more severe disease with invasive and extraintestinal complications.

SEAs due to *Salmonella* infections are exceedingly rare, and the available epidemiologic data on this condition are limited [5]. Diagnosis is notably challenging due to the myriad of potential risk factors and their varying contributions to the disease [6]. Reported risk factors for SEA due to NTS include autoimmune disease, malignancy, diabetes mellitus (DM), sickle cell disease, alcohol use disorder, human immunodeficiency virus (HIV), recent travel to endemic regions, immunosuppression, recent trauma or surgery, and ingestion of contaminated raw foods or water [7–12]. Atypical presentations and the absence of classic predisposing factors in many patients compound this complexity [13]. Early antibiotic treatment combined with urgent surgical intervention is critical in preventing neurological complications. This case report highlights a SEA caused by NTS in a patient with alcohol use disorder, emphasizing the need for a high clinical index of suspicion in patients with risk factors who present with atypical symptoms [3].

## 2. Case Representation

A 49-year-old woman with a medical history of alcohol use disorder presented to the emergency department (ED) with the sudden onset of left flank pain and shortness of breath. One week prior to presentation, she experienced a 3-day illness with fever, chills, and diarrhea. Initial computerized tomography (CT) scan of the abdomen and pelvis revealed nonobstructive nephrolithiasis. Chest CT angiography (CTA) revealed multilevel thoracic spondylosis. A provisional diagnosis of musculoskeletal pain was made. She received ketorolac and methocarbamol and was discharged home due to improvement in symptoms. She returned to the ED 4 days later with progressive, sharp mid-back, left flank, and upper abdominal pain. Associated symptoms included chills, nausea, and abdominal bloating without lower extremity weakness, loss of bladder, or bowel continence. She had no history of intravenous drug use, recent trauma, or steroid spinal injections. On admission, vitals were notable for a temperature of 98.9°F, heart rate of 90 beats per min, and respiratory rate of 18 breaths per min. A systolic murmur along the left lower sternal border was heard on cardiac auscultation. Abdominal examination revealed a soft abdomen with generalized tenderness. Examination of the cervical spine revealed pain with active range of motion in all directions, while reproducible tenderness was noted on palpation of the thoracic and lumbar spine. Cranial nerve examination was unremarkable. Motor strength was 5/5 in all muscle groups, and reflexes were normal and symmetric. Sensory testing revealed intact light touch, pain, temperature, and proprioception. The patient's tandem gait was normal.

**Figure 1:**
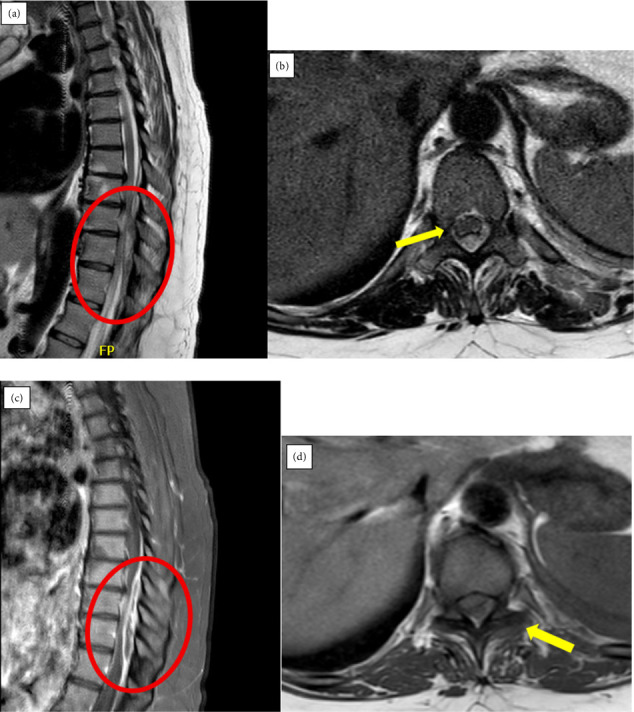
Sagittal (a) and axial (b) T2-weighted MRI images show a dorsal epidural fluid collection (yellow arrow) extending from T8 to T11 (red circle), consistent with an abscess, causing severe thecal sac effacement. Sagittal (c) and axial (d) postcontrast T1-weighted MRI images demonstrate abnormal enhancement in the left T9-T10 facet joint (yellow arrow), with associated effusion, paraspinal extension, and possible osseous erosion (red circle) findings consistent with septic facet arthritis.

Complete blood count revealed white blood cells (WBCs) of 14.2 × 10 × 9/L (77% neutrophils). There were no abnormalities noted on urinalysis. There was no growth on blood cultures. Inflammatory markers were assessed. C-reactive protein (CRP) and erythrocyte sedimentation rate (ESR) were measured on Hospital day 4 and were 40 mm/hr and 16 mg/L, respectively. Transthoracic echocardiography (TTE) was negative for endocarditis. Magnetic resonance imaging (MRI) of the thoracic spine with and without contrast revealed a peripherally enhancing dorsal epidural fluid collection extending from T8 to T11, measuring approximately 0.7 × 1.3 × 6.5 cm. Severe thecal sac effacement was most pronounced at T8-T9 and T9-T10 (Figures 1(a) and 1(b)). Postcontrast T1-weighted sequences demonstrated abnormal enhancement in the left T9-T10 facet joint with associated joint effusion, paraspinal extension, and possible osseous erosion (Figures 1(c) and 1(d)).

Empiric treatment with intravenous vancomycin, ceftriaxone, and metronidazole was begun prior to transfer to a tertiary medical center. In addition, she underwent an emergent T9/T10 decompressive laminectomy and evacuation of an epidural abscess. NTS was isolated from surgical cultures. She had an uneventful recovery and was discharged to complete 8 weeks of oral ciprofloxacin. At the 1-month follow-up visit, she was participating in home physical therapy and reported occasional muscle spasms.

A timeline summarizing the patient's clinical course from initial symptom onset through follow-up is shown in Table 1.

## 3. Discussion

SEA is a severe infection requiring urgent neurosurgical intervention to prevent permanent neurological deficits [1]. SEA caused by *Salmonella* species is extremely rare, with only a few cases reported [8, 9]. We summarized the detailed characteristics of thirteen patients, diagnosed with SEA due to NTS in the literature (Table 2). The majority of the patients were male, and there was a predominance of lumbar involvement.

Among the various reported risk factors for SEA due to NTS, our patient's only identifiable risk factor was alcohol use disorder. Alcohol was shown to have deleterious effects on leukocyte mobilization, phagocytosis, intracellular killing of bacteria, and T helper–1 lymphocyte-mediated cellular response [20]. Other literature suggests alcohol increases the risk of SEA by causing a chronic immunosuppressed state, impaired blood flow, and inducing the inflammatory response [6, 21]. Alcohol often coexists with other risk factors, including diabetes and obesity, further increasing the risk of SEA, as seen in one case reported in Table 2.

Interestingly, SEA can also occur in patients without any identifiable predisposing factors, making atypical cases more challenging to diagnose [22]. The classic triad of fever, back pain, and neurological symptoms is present in only a minority of patients diagnosed with SEA. Most patients tend to present with atypical or nonspecific symptoms, leading to unnecessary diagnostic delays and potentially resulting in neurological deficits. As shown in Table 2, 5 out of 13 reported cases of *Salmonella* SEA presented with atypical symptoms, contributing to challenges in timely diagnosis and treatment [14–17]. Significant delays in diagnosis have been reported in the literature and range from days to months, with 8 weeks being the longest time to diagnosis [23, 24]. We recommend careful history-taking, evaluating risk factors for which a direct role in the causation of SEA can be invoked, such as a prodromal diarrheal illness, as was the case in our patient.

There is no specific protocol for the early diagnosis of atypical SEA, and each case varies, relying heavily on physician judgment based on medical history, physical examination, laboratory tests, and imaging results [3]. Few studies have examined specific strategies for reducing delays in the diagnosis of SEA. Leukocytosis is not consistently present in patients with SEA, occurring in about 60%–80% of cases [15, 18]. Blood cultures are positive in only a proportion of SEA cases, with reported rates ranging from 44% to 60% in the literature [1]. In our review of published cases of *Salmonella* SEA (Table 2), nearly half of the patients [3–5, 15, 19, 25] did not have a positive blood culture, and the diagnosis was established through tissue aspirates or surgical specimens. Similarly, in our case, blood cultures were negative, and the causative organism was identified only from surgical cultures. Therefore, the absence of leukocytosis or positive blood cultures does not definitively rule out SEA. However, inflammatory markers such as CRP and ESR are typically elevated in patients with SEA. The elevation of these markers, especially in those experiencing severe back pain, should raise suspicion for the condition [10]. In our case, ESR was measured on Hospital day 4 and was 23 mm/hr. Although obtained later in the admission and not available during the early diagnostic phase, this finding is consistent with reports that inflammatory markers are typically elevated in SEA.

Once SEA is suspected, a gadolinium-enhanced MRI should be obtained. However, it should be noted that the sensitivity in diagnosing SEA is 91% [23, 24]. It could be nondiagnostic early in the disease course, as seen in one of the patients summarized as follows, where a repeat MRI identified an abscess 8 weeks later. Image-guided biopsy or fluid sampling should be performed to confirm the diagnosis and guide antimicrobial treatment. In some cases, blood cultures may identify the causative pathogen, eliminating the need for invasive sampling. Lumbar puncture is generally contraindicated in patients with suspected SEA due to the potential risk of spreading the infection into the subarachnoid space [11]. Both Alerhand et al. and Tetsuka et al. [16, 26] highlight the limitations of relying on the classic triad and instead advocate for structured diagnostic algorithms. Alerhand's framework is tailored to the ED and prioritizes rapid identification based on risk factors, inflammatory markers, and a low threshold for emergent MRI. In contrast, Tetsuka et al. provide a broader algorithm applicable across care settings, emphasizing the integration of clinical risk assessment, inflammatory markers, and the critical role of early and repeated gadolinium-enhanced MRI. Together, these reviews underscore the importance of algorithmic approaches in reducing diagnostic delays and improving outcomes in SEA.

While gadolinium-enhanced MRI remains the gold standard imaging modality for the diagnosis and follow-up of spinal infections due to its high sensitivity, CT also plays an important complementary role. CT is particularly valuable in the detection and characterization of osseous changes, including vertebral body necrosis and terminal plate destruction, which often develop later in the course of the disease (3–6 weeks after symptom onset). In patients with contraindication to MRI or when bone involvement is suspected, CT can provide critical diagnostic information and guide surgical planning [27–29].

The management of SEA due to NTS involves both surgical and medical therapy. Empiric intravenous antibiotics should be initiated promptly once SEA is suspected, ideally after obtaining blood cultures, as delays in treatment are associated with neurological deterioration (25). Although the 2015 IDSA guidelines recommend fluoroquinolones such as ciprofloxacin for 6–8 weeks as the preferred therapy for *Salmonella* vertebral osteomyelitis and related spinal infections [25], our review of published cases demonstrates that 11 out of 13 patients were treated primarily with ceftriaxone, often in combination with surgical decompression. The frequent use of ceftriaxone may reflect clinician preference for broad-spectrum β-lactam coverage, availability, or concerns about the increasing prevalence of fluoroquinolone-resistant NTS strains and the intracellular persistence of *Salmonella* and its contribution to treatment failure and relapse [30]. We included a case of relapsed SEA, necessitating a switch to different antibiotics and a delay in treatment in Table 2 [31]. Successful treatment typically includes urgent surgical debridement to achieve adequate source control, particularly in patients with neurological compromise and combination with intravenous antibiotics [10]. Radiology-guided drainage may be considered in those who are poor surgical candidates or for small abscesses [7, 18, 31]. Medical therapy alone has been used in selected patients with very poor predictors, such as persons who are unable to tolerate surgery due to extremely poor medical conditions, those with paralysis for > 48–72 h, or if surgery is not indicated for source control [23]. However, medical therapy alone has also been successfully used in patients without neurologic deficits and with a known causative organism with susceptibilities for guiding antibiotic therapy [11, 14]. Close monitoring for signs and symptoms of relapse after completion of antimicrobial therapy is essential for managing SEA.

## 4. Conclusion

SEA is a critical condition with significant morbidity that can be challenging to diagnose early, particularly with atypical presentation. This case report of a 49-year-old woman with a history of alcohol use disorder who developed SEA due to *Salmonella* underscores the importance of maintaining a high index of suspicion in patients with nonspecific symptoms. Timely MRI and prompt neurosurgical consultation were crucial for effective management in this case. Our literature review highlights the diverse manifestations of SEA and the diagnostic difficulties, particularly in patients without classic risk factors. Early diagnosis relies on thorough history-taking, clinical judgment, and appropriate imaging. More extensive studies should be conducted to evaluate the cost-effectiveness of early MRI and the potential benefits of repeat imaging when initial results are nondiagnostic. Developing comprehensive guidelines for managing atypical SEA presentations could improve early diagnosis and outcomes for patients with this rare but serious condition.

## Figures and Tables

**Table 1:** Timeline of clinical course, from initial diarrheal illness through emergency department presentations, MRI diagnosis, surgical intervention, discharge, and 1-month follow-up.

Date/timeline	Event/clinical course
Day 7	Initial 3-day febrile illness with diarrhea and chills
Day 0	Emergency department visit: flank pain, shortness of breath. CT abdomen/pelvis: nephrolithiasis. Chest CTA: thoracic spondylosis. Diagnosed as musculoskeletal pain, discharged
Day 4	Returns to ED with worsening back and flank pain, nausea, bloating. Exam: spine tenderness, no neurological deficit
Day 5	MRI thoracic spine: dorsal epidural abscess T8–T11 with severe canal stenosis. Transferred to the tertiary center
Day 6	Emergent T9-T10 decompressive laminectomy and evacuation of abscess. Intraoperative cultures grew nontyphoidal *Salmonella*
Hospital stay	Empiric IV vancomycin, ceftriaxone, metronidazole ⟶ switched to oral ciprofloxacin for 8 weeks
Discharge	Patient discharged home on oral antibiotics, arranged physical therapy
1-month follow-up	Recovering well, occasional muscle spasms, active in physical therapy

**Table 2:** Summary of cases of nontyphoidal *Salmonella* spinal epidural abscess.

Age/sex	Risk factor/associated condition	Clinical presentation	Clinical presentation at the time of diagnosis	Time to diagnosis	MRI finding/site	Inflammatory markers	Source of positive culture	Antimicrobial regimen	Surgical treatment	Outcome	Reference
69/F	SLE on remission	Back pain, without fever	Lower limb weakness, paraplegia, fever, hypotension	3 days	T7 epidural abscess	CRP 22 mg/dL, ESR 60 mm/h	Blood culture, *S. enteritidis*	Vancomycin, cefepime	Open drainage	No neurological deficit	Araujo et al. [5]
17/M	Recent travel (Kenya and Tunisia)	Diarrhea and malaise for 2 weeks	Worsening lower back pain and malaise	8 weeks	L1-L2 intradiscal abscess	Normal CRP, ESR 44 mm/h	Biopsy of the lumbar spine, *S. enteritidis*	Ceftriaxone, cotrimoxazole for 12 weeks	No surgical intervention	No neurologic deficit at 4-month follow-up	Humayun et al. [14]
59/F	Rheumatoid arthritis treated with tocilizumab	Neck pain, upper extremities paresthesia	Neck pain, which worsened 1 week after admission	On admission	C4 and C5 epidural abscess and discitis	CRP < 0.5 mg/dL, ESR 7 mm/h	Aspirate culture, *S. enteritidis*	6 weeks of ceftriaxone	Debridement, C4-5 vertebrectomy, and C3-C6 fusion	No recurrence	Douglass et al. [15]
54/M	HIV/AIDS	Lower extremities weakness, urinary retention, and fecal incontinence	Unchanged	2-3 days	Osteomyelitis/discitis at T2/T3, T5/T6, T1-T8, circumferential epidural phlegmon at T4	Not done	Wound cultures, *S. enterica serovar Agbeni*	Levofloxacin for 8 weeks	No surgical intervention	Ambulatory difficulty requiring subacute rehabilitation	Bengrad et al. [11]
65/M	Type 2 diabetes mellitus	Polyuria, polydipsia, back pain, and dysuria	Persistent fever	3 weeks	L5/S1 discitis/osteomyelitis and epidural abscess	CRP 291 mg/L, ferritin 718 μg/L	Blood culture, *Salmonella* spp.	Fusidic acid and ertapenem for 8 weeks	L3/L4 laminectomy	Not documented	Farrar et al. [16]
50/M	No pertinent risk factor	Back and right hip pain, fever	Fever, right hip and low back pain, weakness	2 weeks	L2-L3 osteomyelitis and discitis with eccentric disc bulge	CRP 25.2 mg/dL, procalcitonin 11.11 ng/mL	Blood cultures, *Salmonella enterica*	Ceftriaxone 2 g for 4 weeks, then levofloxacin 750 mg daily	CT-guided aspiration	No neurological deficits	Mousselli et al. [4]
68/M	Alcohol use disorder, diabetes,	Fever, shortness of breath, and influenza-like symptoms	Fever, severe back pain	4 days	T8–T10 epidural abscess	Not performed	Blood cultures, *S enterica spp*, *serovar Dublin*	Long-term unspecified antibiotics	Unspecified intervention	No neurological deficit	Avenatti et al. [17]
54/M	No pertinent risk factor	Back pain following a fall from an 8-foot ladder	Worsening lower back pain and bilateral lower extremity weakness in both L5 and S1 distributions	5 weeks	Discitis/osteomyelitis and epidural abscess at L5–S1	Not performed	Tissue and wound cultures, *S. enterica serovar Agbeni*	8 weeks IV ceftriaxone	Decompression, laminectomy and discectomy	No neurological deficit at follow-up	Dahlberg et al. [3]
27/M	Sickle cell disease, splenectomy	Low back pain	Fever, worsening tachypnea and cough	3 days	T5-T6 spondylodiscitis, T4–T7 multiloculated intraspinal epidural abscess	CRP 138 mg/L, procalcitonin 0.17 ng/mL	Blood, sputum, epidural fluid cultures, *Salmonella* (non- Typhi)	Ceftriaxone for 4 weeks, followed by TMP-SMX (2 weeks)	Decompression, posterior T4–T7 laminectomy, and drainage of epidural abscess	No neurological deficit	Elnour et al. [9]
53/M	Well-controlled DM	Fever, acute back pain	Persistent acute back pain, fever, and numbness	3 days	L1 to L5 epidural abscess, osteomyelitis, and psoas abscess	CRP 29.0 mg/	Blood culture *Salmonella Altona* (O8:r:z6)	Ceftriaxone and ciprofloxacin for 6 weeks followed by oral ciprofloxacin for 2 weeks	Drainage at Days 11 and 34 of hospitalization	No neurological deficit	Hirai et al. [18]
54/F	None	Fever, low back pain, paresthesia, and progressive difficulty standing	Confusion, loss of bowel and bladder control	Unspecified	T2, T12-L3 epidural collection and thickness at L2-L3	Procalcitonin 10.7 ng/m	Blood, epidural fluid, urine cultures, *S. enteritidis*	Ceftriaxone for 6 weeks, followed by PO ciprofloxacin for an undetermined duration	Right L1-2, L2-3, and L3-4 hemilaminectomies	No neurological deficit	Majid et al. [12]
50/M	Myelofibrosis	Fever, diarrhea, and severe low back pain	Increased bilateral tone, brisk reflexes, bilateral downgoing plantars	2 weeks	Spondylodiscitis at L4-5 with multiple paraspinal abscesses	Not performed	Blood culture, *Salmonella*	Ceftriaxone for 8 weeks	Laminectomy and aspiration	No neurological deficit	Fareed et al. [10]
62/M	None	Back pain	Persistent back pain	2 Months	Paravertebral abscess and spondylitis on repeat MRI	ESR 49 mm/hr, CRP 0.34 mg/dL	Abscess aspirate, *Salmonella* group D	Ceftriaxone and ciprofloxacin for 13 weeks	Drainage	No neurological deficit at	Chao et al. [6]
56/M	Diabetes	Back pain radiating to the anterior chest wall, fever, paraparesis, sphincteric urgency	Fever, low back pain, paresthesia, ambulatory difficulty, confusion and progressive weakness	On admission	Edematous T2- T4 vertebral bodies, T3-4 disk space abscess	CRP 19 mg/dL, ESR 95 mm/hr	Abscess cultures	Ceftriaxone for 6 weeks	T3-4 costotransversectomy	No neurological deficit	Abdullah et al. [19]

## Data Availability

All data supporting this case are included within the article.

## References

[1] Reihsaus E., Waldbaur H., Seeling W. (2000). Spinal Epidural Abscess: A Meta-Analysis of 915 Patients. *Neurosurgical Review*.

[2] Khoo H. W., Chua Y. Y., Chen J. L. (2016). Salmonella typhi Vertebral Osteomyelitis and Epidural Abscess. *Case Reports in Orthopedics*.

[3] Dahlberg R. K., Lyvers M. E., Dahlberg T. K. (2018). Diagnostic Quandary: Salmonella Agbeni Vertebral Osteomyelitis and Epidural Abscess. *Case Reports in Orthopedics*.

[4] Mousselli M., Chiang E., Frousiakis P. (2022). Epidural Phlegmon and Iliopsoas Abscess Caused by Salmonella enterica Bacteremia: A Case Report. *International Journal of Surgery Case Reports*.

[5] de Araujo D. B., Daolio L., Szajubok J. C., Araujo N. C., Chahade W. H. (2012). Epidural Abscess due to Salmonella Enteritidis in a Patient With Systemic Lupus Erythematosus. *Lupus*.

[6] Chao D., Nanda A. (2002). Spinal Epidural Abscess: A Diagnostic Challenge. *American Family Physician*.

[7] Hsu T. L., Yang C. J., Pao J. L. (2022). Salmonella Spondylodiscitis and Epidural Abscess Successfully Treated With Unilateral Biportal Endoscopic Discectomy and Debridement: A Rare Case Report. *Journal of International Medical Research*.

[8] Yavuz D., Murat D., Cevdet Y. (2006). Spondilodiscitis and Lumbar Epidural Abscess Occurring After Orthopedic Epidural Anesthesia: A Case Report. *Turk Neurosurg*.

[9] Elnour S., Hashim M., Ibrahim H. (2022). Disseminated Non-Typhoidal Salmonella Infection With Salmonella Pneumonia and Vertebral Osteomyelitis in Sickle Cell Disease: A Case Report. *IDCases*.

[10] Fareed S., Nashwan A. J., Abu Jarir S. (2017). Spinal Abscess Caused by Salmonella Bacteremia in a Patient With Primary Myelofibrosis. *American Journal Case Reports*.

[11] Berngard S. C., Miller M. (2013). Salmonella Spinal Infection: A Rare Case in a Patient With Advanced AIDS. *Journal of the International Association of Physicians in AIDS Care*.

[12] Majid R., Vishal D., Al Obaidi M., Elliott F. (2018). Salmonella Enteritidis Concurrent Spinal Epidural Abscess, Urinary Tract Infection and Endocarditis in an Immunocompetent Host: Case Report and Review of the Literature. *Journal of Tropical Diseases*.

[13] Feng Z. Y., Guo F., Chen Z. (2014). Literature Review and Clinical Presentation of Cervical Spondylitis due to Salmonella Enteritidis in Immunocompetent. *Asian Spine Journal*.

[14] Humayun M. A., Richardson T., Brooks A. (2016). Fever of Unknown Origin in a Patient Initially Presenting With Traveller’s Diarrhoea. *BMJ Case Reports*.

[15] Douglass E., Mondy K., Huth R. G. (2016). Salmonella Epidural Abscess in a Patient With Rheumatoid Arthritis Treated With Tocilizumab. *Infectious Diseases in Clinical Practice*.

[16] Farrar H., Abbey A., Patel V., Nair R. (2015). Osteomyelitis, Discitis, Epidural and Psoas Abscess Secondary to Salmonella enterica in a Man With Diabetes Mellitus and Newly Diagnosed α-thalassaemia Trait. *BMJ Case Reports*.

[17] Avenatti E., Iafrati M. D., Patel V., Little S. H., Pandian N. G., Ianchulev S. A. (2017). Acute Aortic Syndrome-More in the Spectrum. *Journal of Cardiothoracic and Vascular Anesthesia*.

[18] Hirai N., Kasahara K., Yoshihara S. (2020). Spinal Epidural Abscess Caused by Non-Typhoidal Salmonella: A Case Report and Literature Review. *Journal of Infection and Chemotherapy*.

[19] Abdullah S. H., Ata O. A., El-Adwan N. (2008). Thoracic Spinal Epidural Abscess Caused by Salmonella typhi-Case Report: Case Report. *Neurologia Medico-Chirurgica*.

[20] Oki M., Ueda A., Tsuda A., Yanagi H., Ozawa H., Takagi A. (2016). Salmonella enterica Serotype Enteritidis Vertebral Osteomyelitis and Epidural Abscess Complicated With Meningitis. *Tokai Journal of Experimental & Clinical Medicine*.

[21] Kido G. R. (2024). Epidural Abscess. *Orthopedie Traumatologie*.

[22] Vilke G. M., Honingford E. A. (1996). Cervical Spine Epidural Abscess in a Patient With No Predisposing Risk Factors. *Annals of Emergency Medicine*.

[23] Alerhand S., Wood S., Long B., Koyfman A. (2017). The Time-Sensitive Challenge of Diagnosing Spinal Epidural Abscess in the Emergency Department. *Internal Emergency Medicine*.

[24] El Sayed M., Witting M. D. (2011). Low Yield of ED Magnetic Resonance Imaging for Suspected Epidural Abscess. *The American Journal of Emergency Medicine*.

[25] Berbari E. F., Kanj S. S., Kowalski T. J. (2015). 2015 Infectious Diseases Society of America (IDSA) Clinical Practice Guidelines for the Diagnosis and Treatment of Native Vertebral Osteomyelitis in Adultsa. *Clinical Infectious Diseases*.

[26] Tetsuka S., Suzuki T., Ogawa T., Hashimoto R., Kato H. (2020). Spinal Epidural Abscess: A Review Highlighting Early Diagnosis and Management. *JMA Journal*.

[27] Román G. C. (2014). Tropical Myelopathies. *Handbook of Clinical Neurology*.

[28] Miyoshi I. C., de Toledo A. H. N., Pereira F. V. (2023). Infectious Myelitis. *Seminars in Ultrasound, CT and MRI*.

[29] Bhattacharyya S., Bradshaw M. J. (2021). Infections of the Spine and Spinal Cord. *Continuum*.

[30] Centers for Disease Control and Prevention (2019). Drug-Resistant Nontyphoidal Salmonella: Threat Level Serious. *Antibiotic Resistance Threats in the United States*.

[31] Fujii M., Shirakawa T., Shime N., Kawabata Y. (2020). Successful Treatment of Extensive Spinal Epidural Abscess With Fluoroscopy-Guided Percutaneous Drainage: A Case Report. *Clinical Reports*.

